# “When You Move You Have Fun”: Perceived Barriers, and Facilitators of Physical Activity From a Child's Perspective

**DOI:** 10.3389/fspor.2022.789259

**Published:** 2022-03-07

**Authors:** Sarah Nally, Nicola D. Ridgers, Alison M. Gallagher, Marie H. Murphy, Jo Salmon, Angela Carlin

**Affiliations:** ^1^Centre for Exercise Medicine, Physical Activity and Health, Sports and Exercise Sciences Research Institute, Ulster University, Newtownabbey, United Kingdom; ^2^School of Exercise and Nutrition Sciences, Institute for Physical Activity and Nutrition (IPAN), Deakin University, Geelong, VIC, Australia; ^3^Nutrition Innovation Centre for Food and Health (NICHE), Biomedical Sciences Research Institute, University of Ulster, Coleraine, United Kingdom

**Keywords:** children, health, qualitative—quantitative analysis, school, physical activity

## Abstract

In Northern Ireland (NI), many children do not meet the recommended levels of physical activity (PA). To reduce the prevalence of physical inactivity and associated health conditions, it is important to understand the influences on children's PA, which in turn has the potential to inform future intervention design. The purpose of this formative study was to examine the current views, barriers, facilitators, experiences, and perceptions of children in relation to PA in the classroom, school, and home environments, and to assess the acceptability of components for a school-based intervention. Write and draw tasks and semi-structured focus groups (*n* = 10) were conducted with 50 children aged 7–9 years (22 boys, 28 girls) from six primary schools. Focus groups were recorded, transcribed, and analyzed thematically. Pen profiles were constructed from the transcripts in a deductive manner and represent key emergent themes. Results indicated that children's perception and knowledge of PA was mainly structured and sport-based, while some referred to fun, play and health. Fun, social support and outdoor activity were identified as key facilitators. Barriers included parental restrictions, lack of time and space in the different environments. The acceptability of intervention components was examined, children recognized the potential benefits of additional movement in the classroom, but opinions differed on the sit-to-stand desks. Findings contribute to a more detailed understanding of children's perceptions of context specific PA, the barriers they face, in addition to factors that support them to lead a physically active lifestyle, which may inform future PA promotion strategies.

## Introduction

Developing effective health promotion strategies targeting primary school-aged children is necessary to reduce the future burden of preventable non-communicable diseases (Janssen and LeBlanc, 2010). Regular physical activity (PA) provides children with broad-ranging physical and psychological health benefits (Poitras et al., 2016), including improved cardiometabolic health, academic performance (Rasberry et al., 2011), cognitive function, and prosocial behavior (Donnelly et al., 2016; Sampasa-Kanyinga et al., 2020). According to the World Health Organization (WHO), a major public health concern is the increasing number of children who fail to meet the current PA guidelines of an average of 60 min/day of moderate- to –vigorous-intensity aerobic PA across the week (Bull et al., 2020; Guthold et al., 2020). This issue is particularly prevalent among primary-school children in Northern Ireland (NI), who are least likely to meet the recommended levels of PA, when compared with other countries in the United Kingdom (UK) (Griffiths et al., 2013) and in Europe (Verloigne et al., 2016). The Children's Participation and Physical Activity Study found that only 20% of primary school children in NI meet PA guidelines (Woods et al., 2019). Research suggests that moderate-to-vigorous physical activity (MVPA) decline begins in early to mid-childhood (Farooq et al., 2020). There are also concerns about coexisting sedentary behavior (SB) in children, specifically screen-based activities like television viewing, which has unfavorable effects on various health indicators, independent of PA (Tremblay et al., 2011).

Schools have been identified as an important setting to promote PA (Neil-Sztramko et al., 2021) and reduce SB (Hegarty et al., 2016), with the WHO specifying that the principle of a whole-of-school approach should be applied (World Health Organization, 2018; Milton et al., 2021). Children spend a significant amount of their waking time at school (Nickel et al., 2021) and many schools have the facilities, equipment, and large amount of contact time to deliver PA interventions. Timing of PA program implementation is important (Milat et al., 2015), teaching staff must not feel over-burdened, and children should not lose out on class time (Day et al., 2019). Several systematic reviews examine efficacy trials provide evidence of a slight increase of time spent in PA for children exposed to school-based interventions (Kriemler et al., 2011; Dobbins et al., 2013: Nally et al., 2021). Neil-Sztramko and colleagues found school-based PA interventions do not result in meaningful changes in MVPA, however, these findings should be interrupted with caution as there was a high degree of heterogeneity across all studies (Neil-Sztramko et al., 2021). The out-of-school period offers additional opportunities for family-based PA. Parents can serve as role models through their parenting practices (Maric et al., 2020), sources of support, and gatekeepers through their control over children's opportunities for PA (Lindsay et al., 2006; Mitchell et al., 2012). Xu et al. (2015) found that there was a positive association between parental encouragement and support with children's PA. Additionally, parents also govern the home environment and can impact PA behavior through the provision of opportunities to be physically active (e.g., accessibility of play and sports equipment) (Maitland et al., 2013).

Despite large-scale quantitative research being able to assess the direction and strengths of the trends of children's participation in PA, they are unable to explain the reasons why children maintain or cease PA. Despite years of such intervention research, increases in PA remain both modest and short term (Mitchell et al., 2012; Craike et al., 2018). There is now widespread recognition that health behaviors are difficult to change (Sheeran et al., 2016). As such, PA interventions have shifted focus to include the factors that influence these behaviors beyond individual choice alone. The socioecological model recognizes that efforts to change health behaviors are more likely to be successful when multiple levels of influence including individual, social, environmental and policy levels are addressed at the same time. To be successful, future intervention development needs to address these multiple influences on behavior, including the family and home setting (Sallis, 2001). The evidence base is not as extensive, as research to date designed to increase PA in children and inform intervention design has largely underrepresented children's perceptions (Noonan et al., 2016) and has been limited to singular qualitative methods that omit children's verbal ability and interaction preference (Morgan et al., 2002). There is a paucity of data analyzing PA barriers and facilitators in the school setting, particularly from the perspective of children aged 7–9 years. Previous literature in this age group examines children's perceptions of obesity prevention (Clarke et al., 2015; Hesketh et al., 2017), not PA.

*Transform-Us!* was designed in response to the aforementioned low levels of PA and high levels of SB. It is a multi-component school- and family-based intervention which was developed through a series of extensive iterative work in Australia (Salmon et al., 2011). *Transform-Us!* aims to increase children's PA and decrease SB across the school day incorporating a mixture of educational, pedagogical, behavioral, and environmental approaches to integrate movement into everyday class lessons and recess/lunchtime. Family-based components comprise of newsletters and homework assignments containing family-based activities for parents to complete with their children. The study is based on elements from the social cognitive theory (Bandura, 1986), theory of planned behavior (Rachlin, 1989) and behavioral choice theory (Bronfenbrenner, 1986). Although the *Transform-Us!* intervention has proved very promising as a multi-component program (Carson et al., 2013; Yildirim et al., 2014) and is in the final stages of longer-term evaluation in Australia (under review), we do not have similar multi-component programs which have been subjected to rigorous evaluation within NI. Several local contextual differences between the Australia and NI, in particular the educational system, social and environmental influences on PA and policy level differences mean that whole scale replication would be inappropriate. Formative research is important in the development and implementation of the intervention, as it identifies specific behaviors, the determinants of these behaviors and collects as much information as possible to assist in forming the intervention aims and objectives (Bleijenberg et al., 2018).

A more detailed understanding of NI children's perceptions of context specific PA, the participation barriers they face, in addition to factors that support them to lead a physically active lifestyle may inform future PA promotion strategies. This exploratory study used a formative assessment to inform the adaptation of a pre-existing PA and SB intervention *Transform-Us!* This study aimed to use a combination of qualitative techniques to explore children's current views, experiences and perceptions of classroom, school, and family-based PA. It was envisaged that the contextual information gathered from this study will provide valuable insights into the connotations children ascribe toward PA and inform the subsequent intervention design of future PA promotion and SB reduction strategies targeting primary-school aged children.

## Materials and Methods

Reporting of this study was guided by the consolidated criteria for reporting qualitative research (COREQ, Tong et al., 2007) (Supplementary Table 1). Ethical approval was granted by the University Ethics Committee (REC/20/0033), and informed written consent and assent was obtained from parents and participants prior to participation. Data collection took place between November 2020 and April 2021, during the second wave of the COVID-19 pandemic. Schools in NI had been closed during the period of March to August 2020 due to a period of ‘lockdown' through public health legislation.

### Recruitment

Participants were recruited from a convenience sample of primary schools spanning a range of socio-economic areas and from both rural and urban areas of NI. Ten primary schools were invited to take part in the study via e-mail/telephone. Eight primary schools agreed to participate in the study. Following school principal consent, information packs containing child and parent information sheets and consent forms were sent to parents/guardians of pupils in Primary 4 (7–8 years old) and/or Primary 5 (8–9 years old). The pupils who returned written assent forms and consent forms from parents/guardians were eligible to participate. The participants did not receive an incentive or compensation for taking part in the study. Prior to the visit, all the pupils were informed about the purpose and procedure of the study. Participants were told that they could withdraw from the study anytime.

### Data Collection

There were two complimentary qualitative methods used in the formative study: a write and draw activity and focus groups exploring children's perceptions of PA and experiences in greater depth. Previous studies have shown that a write and draw activity can provide children with greater control over their expression and the drawings themselves are visual drawings that directly represent children's perspectives and/or experiences (Knowles et al., 2013; Noonan et al., 2016). The drawing task is also appropriate for younger children as it can demonstrate thinking at their own levels of cognitive development (Knowles et al., 2013). Likewise, focus groups are an effective method to explore the ideas and perspectives of children (Gibson, 2007). Using a combination of qualitative research techniques, including sustained interest, enabled the data-generation process to be fun and engaging for participants, prevented bias arising from the overreliance of one method, and served to triangulate and cross-check data (Punch, 2002). Other than participant age, there were no specific inclusion criteria employed. Data collection was facilitated by the first author, in the school's assembly hall or in an outdoor classroom where participants and researcher alike could be overlooked but not overheard. By interviewing children in a group setting at school, we aimed to create a friendly and safe space which would encourage children to share their ideas and thoughts (Adler et al., 2019).

#### Write and Draw Activity

The write and draw activity was completed prior to the focus groups and prior to break time (i.e., morning recess) to reduce the influence recent experiences may have on participants' thoughts and perceptions. The write and draw activity was double sided and contained five sections (Supplementary Material 2). For the purpose of this study, children were asked to express their perceptions of PA visually, and independently, (i.e., not completed in conjunction with peers) during class time in each school. To minimize distraction from the task, no written instructions were provided (Knowles et al., 2013). The first author (SN) facilitated the activity and separately engaged with the children in informal conversations to answer any questions. The first author refrained from providing any feedback.

#### Focus Groups

To further triangulate the data and ensure the credibility and dependability of the findings, participants were invited to express their perceptions of PA through discussions via focus groups. A semi-structured topic guide standardized discussions between subjects and allowed for participants to respond freely whilst also ensuring key topics were covered in detail to allow a degree of comparability across the transcripts (Punch and Graham, 2016). The topic guide was derived from reviewing the existing literature in relation to PA and were designed to be exploratory. The questions focused on the current views, barriers, facilitators, experiences, and perceptions of children in relation to PA in the classroom, school, and home environments, and aimed to evaluate and assess the acceptability of components used in *Transform-Us!* for a school-based intervention (topic guide available in Supplementary Material 3). The topic guide was pilot tested with a group of four primary-school children, from a school that was not included in this study, to ensure that questions were deemed appropriate for this age group. Probing questions were used throughout the focus group to facilitate discussion (Elo et al., 2014).

The first author (female Ph.D. candidate holding an MRes, trained in running focus groups) facilitated separate semi-structured focus groups that included 4–6 children (10 focus groups, *n* = 50). To reduce the potential power imbalance that may arise when an adult facilitates a children's focus group, it was made clear that the moderator was not a teacher, there were no right or wrong answers, and the children were free to express their own opinions (Heary and Hennessy, 2002; Morgan et al., 2002). First names were used to moderate the hierarchical adult-child relationship. Following an explanation of the procedure, verbal assent was obtained from all participants. At the beginning of the focus group, participants were informed that the definition of “physical activity” was any movement of the body that uses energy, and this included all forms of sport, physical education (PE), indoor and outdoor play, adventurous activities, active travel, and normal activities such as using the stairs, doing housework and gardening. The questions asked during the focus groups and the PA definition used were purposely kept broad to capture what types of PA and settings were meaningful from the perspective of the participants. A short icebreaker was used at the beginning of the focus group to help children feel comfortable and relaxed. Photographs presenting a range of active and inactive activities were used to encourage discussion amongst children, having previously been shown to be an effective method (Hesketh et al., 2005). Examples of the activities included children skipping outside, watching television, and a child brushing the floor. Participants were asked several questions regarding the activities (Supplementary Material 3). Moreover, a photograph of a sit-to-stand desk was used to elicit conversation, ensuring the topic was covered in detail and allowed a degree of comparability across the transcripts (Ereaut, 2002). Open-ended questions starting with “what”, “why” and “how” were posed to participants to stimulate conversation. To maintain the interest and enthusiasm of participants and accommodate short attention spans (Colucci, 2007), each focus group discussion lasted between 16 and 30 (mean 20.11) min. With the participants' permission, the interviews were audio recorded using a Homder digital voice recorder.

### Data Management and Analysis

A form of content analysis was used to analyze the write and draw activity. Text responses for the write tasks were extracted verbatim, while drawings were used to identify additional key themes (e.g., playing sport, social interaction, fun). The following procedure and terminology were used to analyze the questions. Drawings had to be legible. Children's responses to the questions were categorized as a “mark”. A “mark” within a child's drawing referred to an item which could be identifiable as theme. It was possible for one mark to identify more than one theme. For example, the identification of how many “times” a theme was identified (e.g., a child drawing a football game and writing “playing football”) would be counted as two times. Ten write and draw sheets were independently reviewed by a second author (N. R.) and a discussion was had about the findings until a consensus of the themes were reached. Subsequently, the data were then analyzed using pen profiles analysis.

The focus group discussions were audio recorded and transcribed verbatim. The analysis consisted of all together 102 pages of double-spaced transcription. All data were managed in NVivo12 (Version 12.6.0; QSR International Pty Ltd., Victoria, Australia) and analyzed independently by SN. To ensure children were unhindered by researchers' previous views about PA in children, in our study we chose a deductive followed by an inductive approach. Braun and Clarke's phases of thematic analysis were used to inform the coding process once S.N had become familiar with the transcripts (Braun and Clarke, 2019). Transcripts were systematically coded, responses were transcribed anonymously, and key themes were developed. The socio-ecological model was used as an initial framework to initially examine the data. The inductive approach allowed different themes within the broader socioecological model constructs to emerge. Quotes that were considered to represent a similar meaning or pattern were clustered together into potential themes and subthemes. During this process, themes were identified and were broadly reflective of the socio-ecological model (Elder et al., 2007) which has been used in previous research to demonstrate the influence various factors have on children's participation in PA, i.e., intrapersonal, interpersonal, and organizational factors (Hesketh et al., 2017; Martínez-Andrés et al., 2020). Quotations from the transcripts were extracted to further contextualize key findings from the discussions. The second author acted as a “critical friend” by independently reviewing a subsample of transcripts and offering alternative interpretations of the data, encouraging reflection, and challenging the initial thematic structure. The research group critically discussed all steps and outcomes in each phase of the data analysis process until they reached consensus. In case of disagreements and uncertainties, another member was consulted. Furthermore, Merriam and Tisdell (2016) strategies to promote validity and reliability were used to enhance trustworthiness.

The pen profile approach has been utilized in studies examining youth PA (Mackintosh et al., 2011; Ridgers et al., 2012; Noonan et al., 2016; Taylor et al., 2018). Pen profiles are a visual approach for presenting key themes identified during data analysis through combining verbatim quotes taken directly from the transcripts and write and draw to provide further context and insight to the discussions. The authors critically questioned the analysis and interrogated the data, tracking the process in reverse from the pen profiles to the write and data sheets and/or the transcripts, until a consensus had been reached. Thus, methodological rigor, credibility and transferability was reached via verbatim transcription of data and triangular consensus procedure. In some cases, visual illustrations were added to add further context to the data. Triangulation of the analysis occurred through presentation of the pen profiles together with associated verbatim/illustrative material by the research team.

## Results

Data are presented in five pen profiles, and focus on children's perspectives on PA, PA knowledge, PA barriers and facilitators and intervention approaches.

### Write and Draw

**Figure 1:**
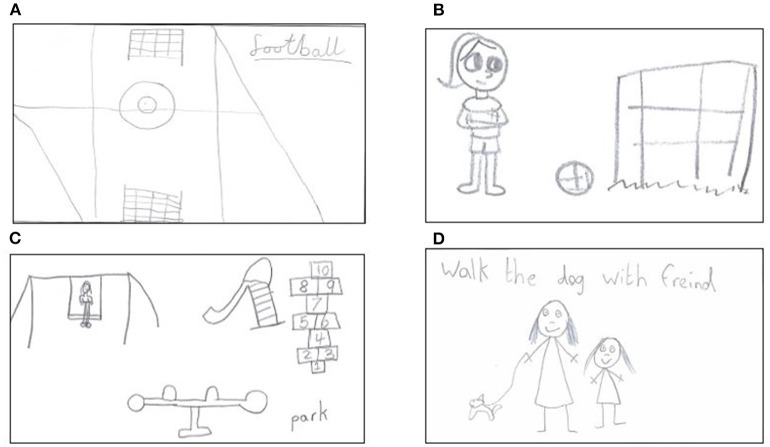
**(A)** Drawing from a boy (ID9) illustrating the football pitch as where he is most likely to engage in physical activity. **(B)** Drawing from a girl (ID34) illustrating what she dislikes about physical activity. **(C)** Drawing from a boy (ID2) illustrating the places where he habitually engages in physical activity. **(D)** Drawing from a girl (ID17) illustrating a walk with her dog and friend.

Fifty primary-school aged children (22 boys, 28 girls) completed the write and draw activity. Blank spaces and answers were due to children not having sufficient time to complete the activity or not providing an answer. A total of 49 participants completed the “what does physical activity mean to you?” section, with all the responses legible. PA was mostly associated with sport-based activity e.g., football (*n* = 10), dancing (*n* = 7) and structured activity e.g., PE (*n* = 8), with participants also defining PA as “sport” (*n* = 17). Furthermore, participants refer to fun (*n* = 6), health (*n* = 6) and play-based activities (*n* = 3). Several reported “*movement*” as their definition of PA. For example, one child wrote that: “*any movement that keeps your health up”* (ID37) and another child wrote: “*it means to me it is sports and help your body. Any sort of movement”* (ID39). Drawings linked to the write and draw activity are presented in Figure 1.

**Figure 2:**
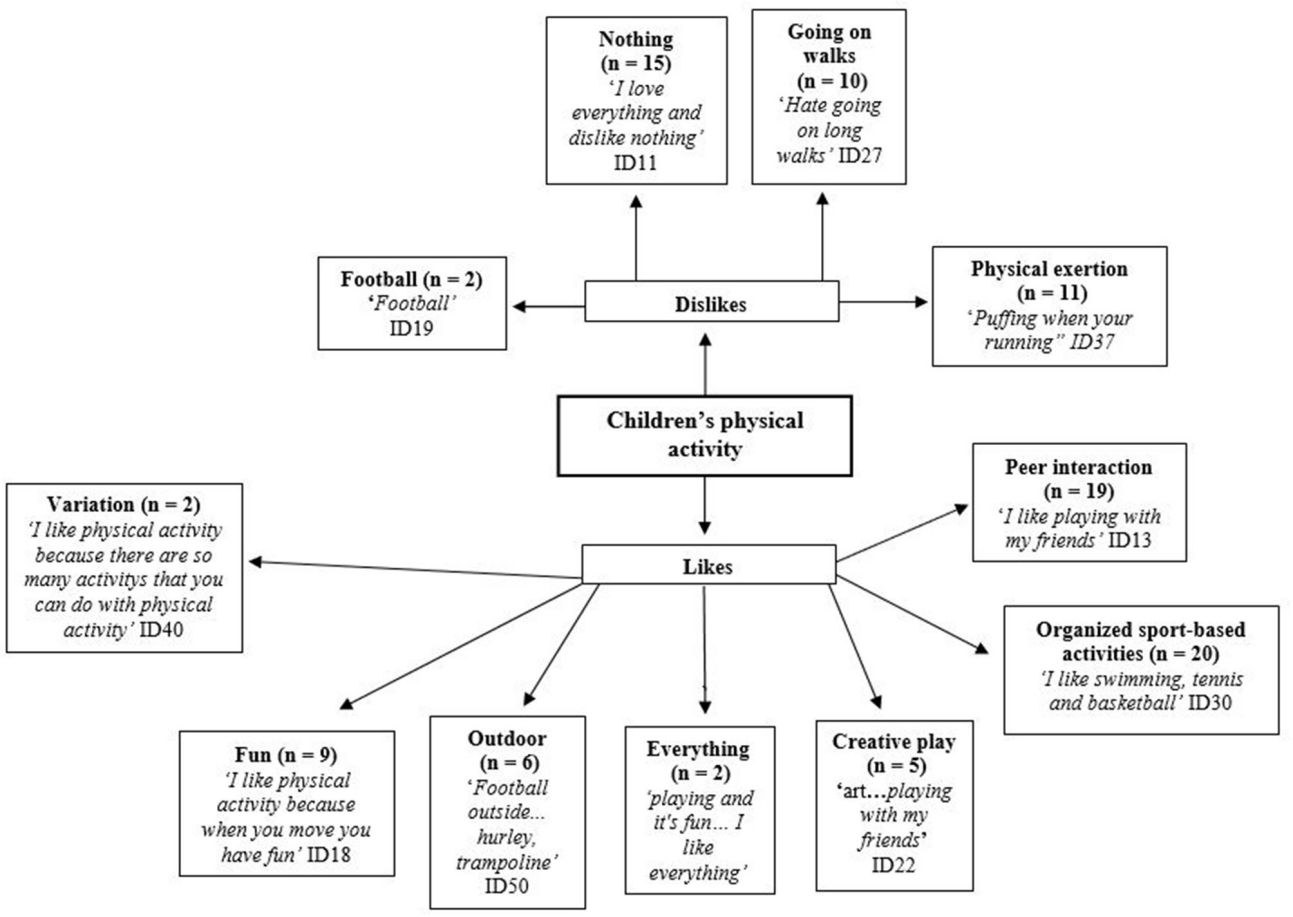
Children's likes and dislikes toward physical activity.

Forty-nine participants completed the “what I like about physical activity” section, and 215 marks on specific themes with two unidentifiable responses were noted within the data analysis. Forty-seven participants completed the “what I dislike about physical activity” section, and 108 marks on specific themes were noted within the data analysis and there were no unidentifiable responses. Table 1 illustrates completion by write or draw section. Figure 2 demonstrates the combined pen profiles of children's likes and dislikes regarding PA. A range of physical activities participated in by the children were described, with the most common being organized sports (*n* = 20) (i.e., football, hockey) cycling, swimming, tennis, and walking. Though the activities undertaken varied across the cohort, a consistent reason for PA participation was for fun (*n* = 9) and for peer interaction (*n* = 19). Many pupils suggested that there was nothing that they disliked (*n* = 15) about PA. Physical exertion (*n* = 11) and going on walks (*n* = 10) were also commonly reported as dislikes.

**Table 1:** Write and draw task completion by section.

**Section**	**Writing in the box**		**Drawing in the box**	
	**Provided**	**Legible**	**Provided**	**Legible**
PA likes	84%	92%	31%	100%
PA dislikes	87%	100%	54%	89%
Place most likely to take part in PA	94%	100%	40%	96%
Types of PA participated outside of school	96%	100%	29%	100%

*PA, Physical activity*.

**Figure 3:**
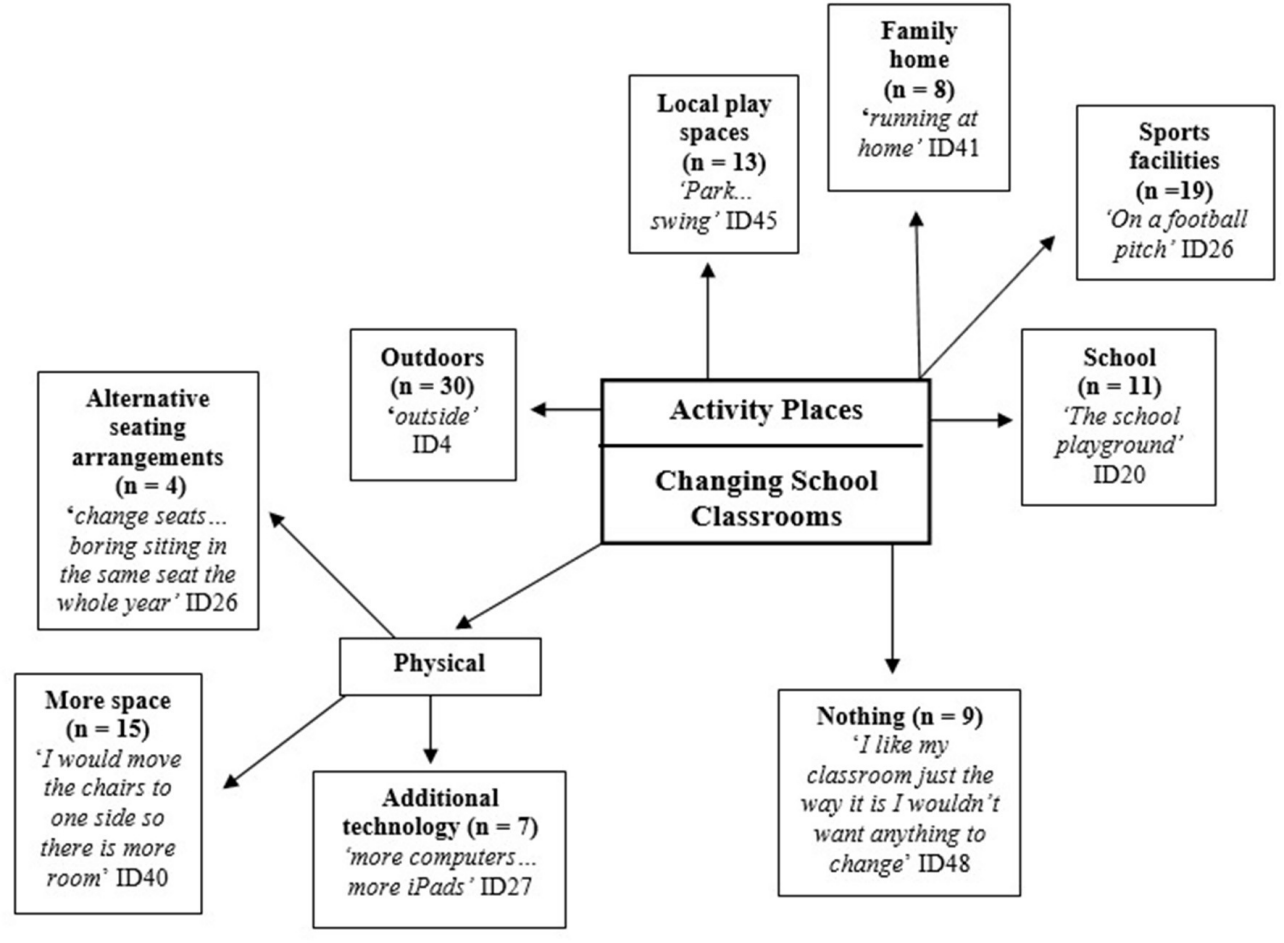
Write and draw activity.

Figure 3 illustrates the composite pen profile which denotes the key themes from the following two questions from the write and draw activity: “if you could change your classroom, what would you change?” and “where are you most likely to take part in physical activity?”. Blank returns were due to insufficient time to complete the task. Fifty participants completed the “if you could change your classroom, what would you change?” section. All the written responses were identifiable and there were 125 marks on specific themes within the data analysis. Physical changes to the classroom were the most requested classroom changes by the children. The “Where are you most likely to take part in physical activity?” section was completed by all the participants (*n* = 50). A total of 138 marks on specific themes within the data analysis were found and data revealed that outdoor (*n* = 30) and sports facilities (*n* = 19) were the commonly reported themes. Forty-eight participants completed the “Over the past week what sort of physical activities did you do outside of school?” section. A total of 172 marks on specific themes within the data analysis were noted, structured sport-based activities (*n* = 28), outdoor play (*n* = 26) and social interaction (*n* = 19) were identified as the highest frequency themes.

### Facilitators of Physical Activity

**Figure 4:**
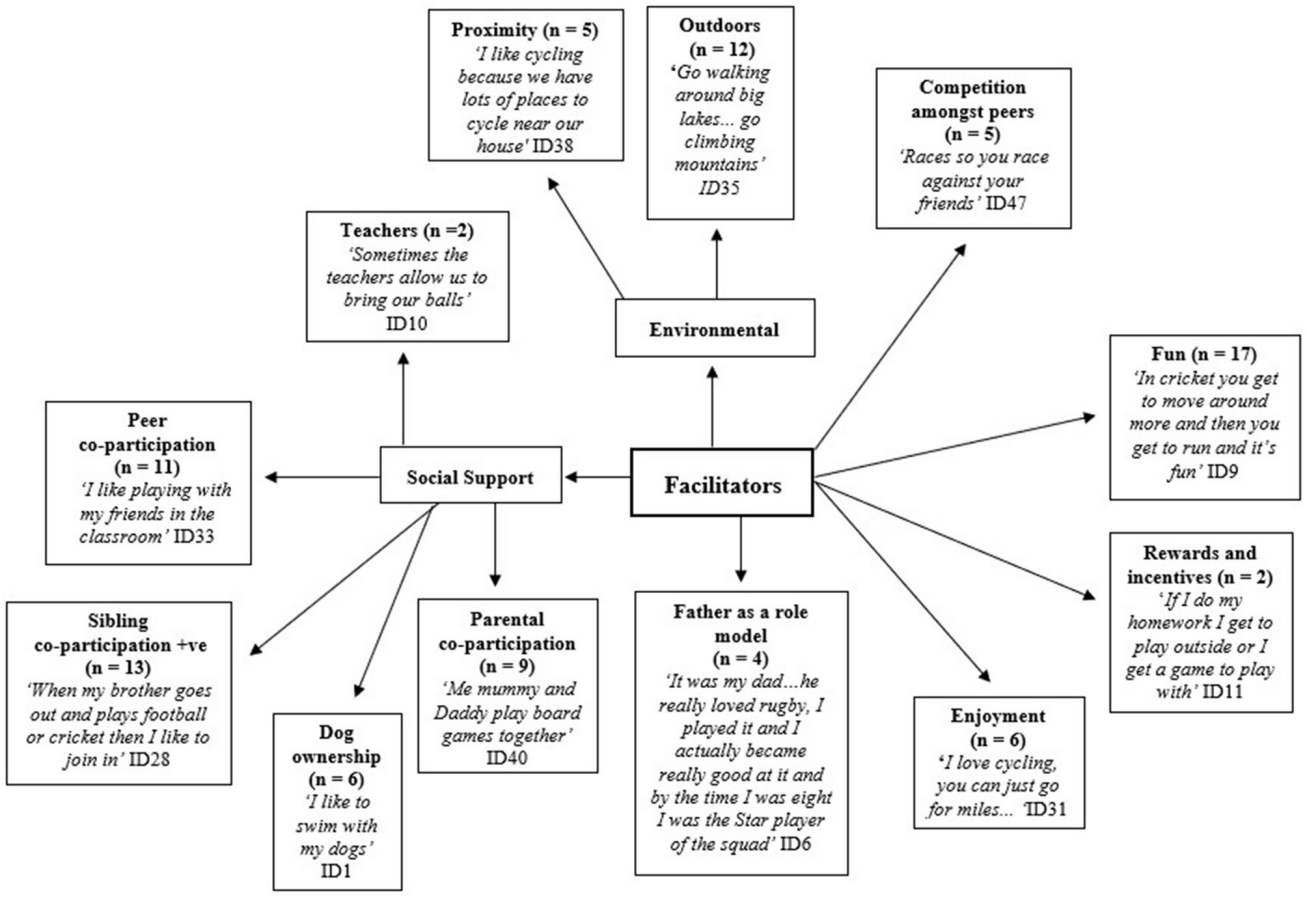
Facilitators to children's physical activity +ve = positive.

Overall, children reported a diverse range of PA facilitators within the school and family environments (Figure 4). One of the primary motivations reported by children was the sense of fun experienced through the social aspect of PA. Children in all focus groups highlighted the importance of social support from their friends, but family members were also noted. Being able to go outside, and enjoyment of PA were also commonly reported facilitators.

Some children expressed a strong desire to exercise, with PA allowing them an outlet from daily routines and the ability to share enjoyment with friends. The competitive and vigorous nature of organized physical activities appeared particularly appealing and enjoyable for many children as they perceived them to be more engaging and beneficial to physical health. Peers also played an important role in influencing participants to be active, with some having a positive experience with their classmates (Figure 4). At school, children noted unstructured and structured PA opportunities as facilitators. Boys tended to mention organized sports, especially football, whereas girls commonly referred to creative play, drawing and art. PE seen as an opportunity practice PA and was described as a structured PA opportunity. At school, children were enthusiastic about the competitive element and challenges occasionally posed amongst peers in PE class or during breaktime. Children specified that family and school play a role in promoting and raising PA awareness. Parental co-participation in the children's activities were also deemed to facilitate activity (Figure 4).

### Barriers of Physical Activity

**Figure 5:**
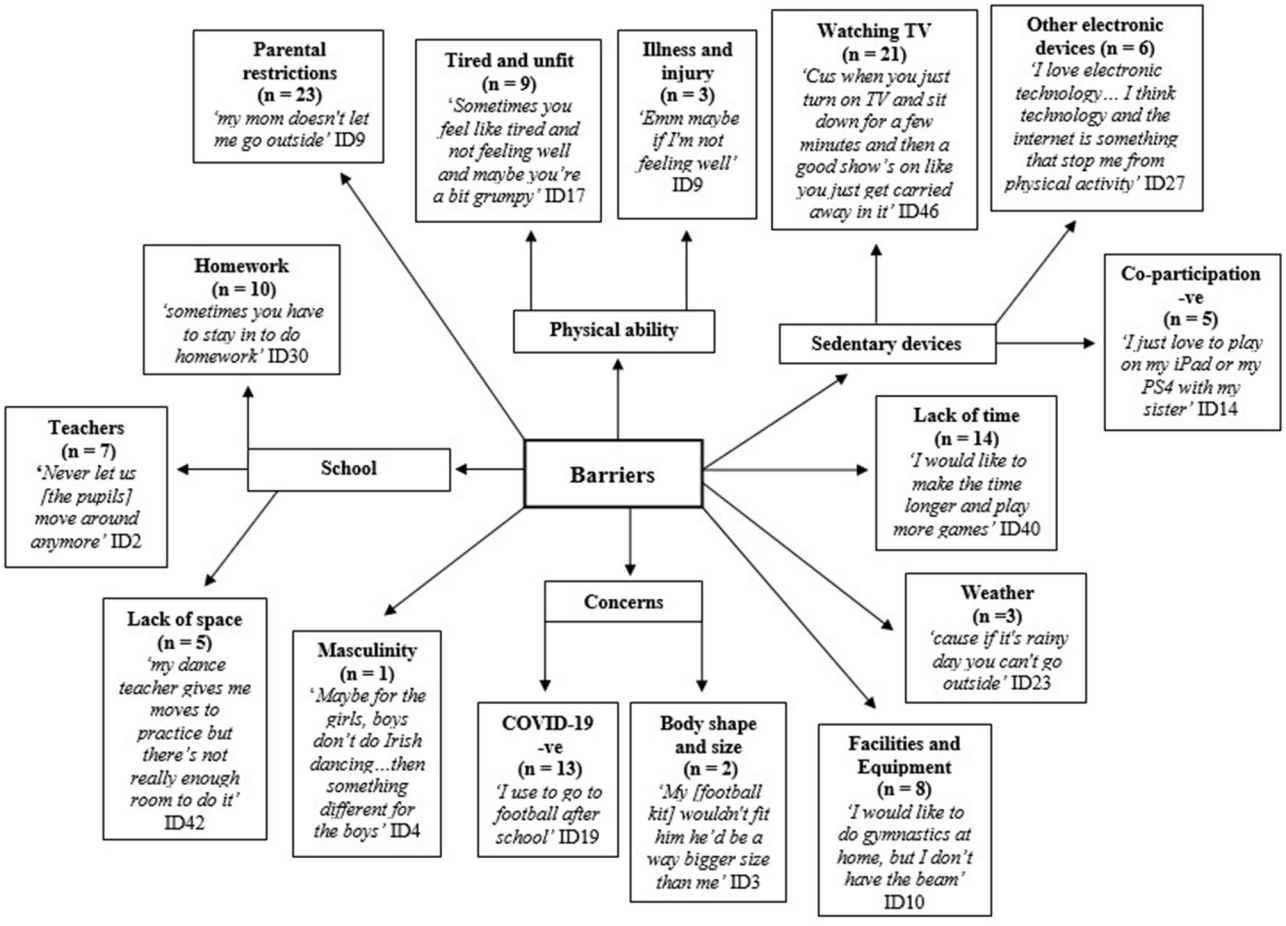
Barriers to children's physical activity -ve = negative.

A range of barriers were reported by the children in relation to PA participation both at home and within school environment (Figure 5). In the home environment, parents restricting PA and being able to freely access and watch TV were key barriers. Using other electronic devices was less commonly reported by these children. Parental restrictions were due to parental time restraints, safety concerns, time, and costs. Children were not allowed go to the park or play near their houses without adult supervision. In contrast, few children spoke about siblings and peers limiting engagement in PA. Children expressed the negative impact weather has on the ability to do PA outdoors.

Regarding the physical environment, children noted that a lack of space, both at school and home, as well as limited/no access to equipment/facilities were barriers. The size of indoor and outdoor space available for play, movement in the classroom and for PE was identified as a challenge in every focus group. The unavailability and the inaccessibility of spaces were also identified as key barriers to children's PA. Due to the lack of space noted inside the sports hall, children favored the outdoor environment for PE at school.

Furthermore, many children stated that they wanted to participate in PA, however, due to lack of discretionary time they were not able to do so. Children also indicated that they do not get time to be active, sometimes owing to external commitments such as homework. Each focus group raised issues of physical ability and explained that these insecurities often prevented them from taking part in PA. Concerns regarding physical ability included tiredness, being unfit, and having an illness or injury. Many children explained that they did not like to participate in PA after school as they were tired. Teachers who limit movement in the classroom were also identified as barriers to maintaining PA.

Children stated that the COVID-19 pandemic was a key barrier to PA participation. Similar to many countries, NI imposed restrictions requiring physical distancing (two meters), and limited community and social gatherings and interactions, sport, and playground and park use. All organized after-school activities were canceled. Thus, most children had to comply with physical distancing, activity constraints and behavior restrictions (Fegert et al., 2020).

### Intervention Approaches

**Figure 6:**
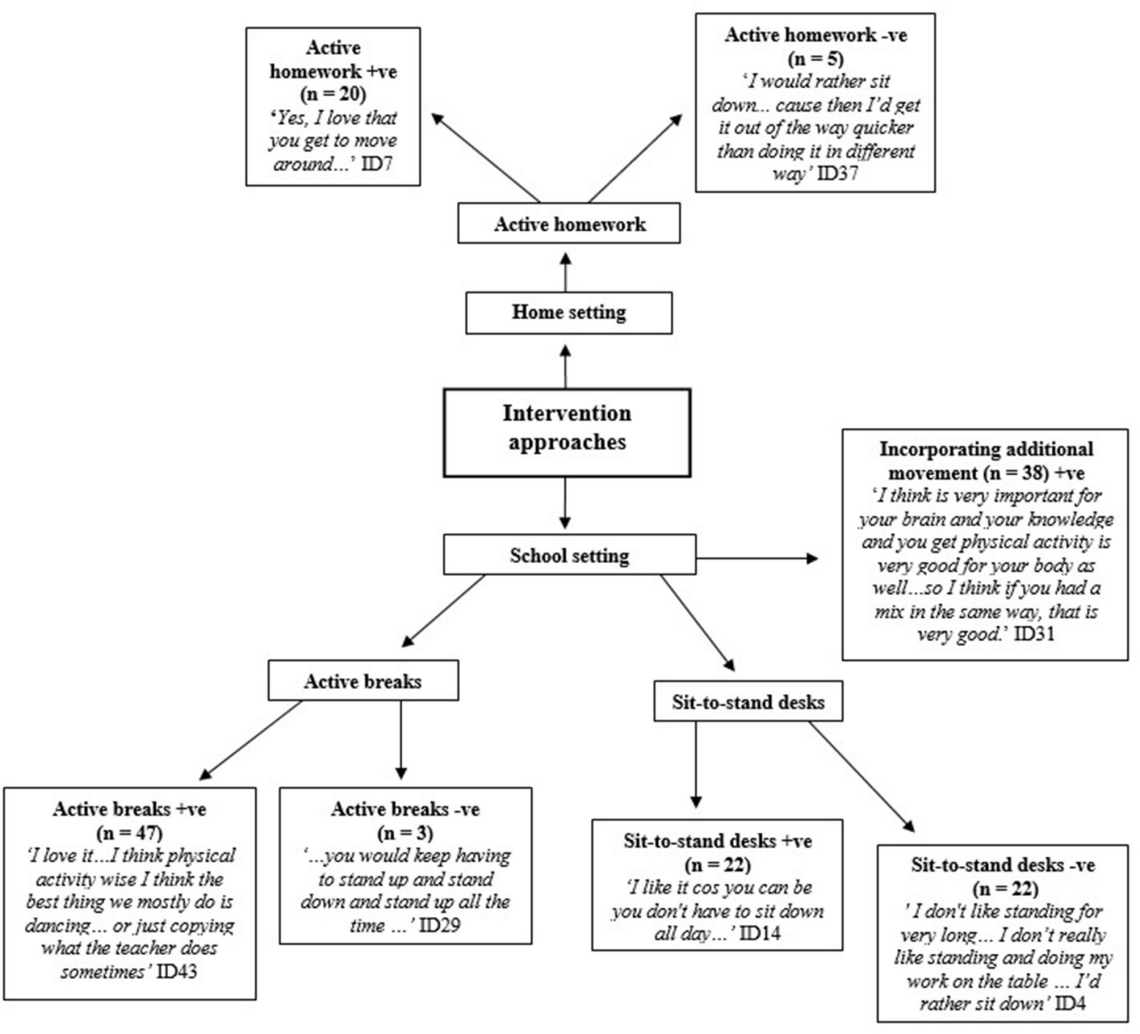
Intervention approaches to tackle children's physical inactivity +ve = positive -ve = negative.

Children discussed a number of intervention components, including active homework, sit-to-stand desks, and active breaks (Figure 6). In each focus group, children stated that they enjoyed the opportunity to be active during the school day. The flexibility and adaptability of active homework appealed to children. The idea of incorporating movement and active breaks into the classroom were perceived as fun. Children were enthusiastic about the additional movement in the classroom component and suggested that it may help them with their academic performance and health.

Children had a mixed response to the sit-to-stand desks. Children were apprehensive regarding the mechanical and logistical side of the sit-to-stand desks. Furthermore, children voiced their concerns regarding the flexibility and adaptability of the desks in the classroom. Additional barriers to the implementation of sit-to-stand desks included time within an intense curriculum, space, and tiredness after prolonged standing. Children noted that they wanted to change the seating and table arrangements to make different patterns to create more space.

## Discussion

The aim of this research was to examine the influences on children's PA current views, experiences, and perceptions of PA in the classroom, school, and home environment and to examine the perceived benefits and barriers of participation. Although previous qualitative research has examined barriers and facilitators to primary-school children being physically active (Brunton et al., 2003; Hesketh et al., 2017), most studies were based in the USA, making their generalizability to the UK setting uncertain. This exploratory study has identified factors that facilitate and inhibit children's participation in PA from the child's perspective and has provided important considerations for adapting *Transform-Us*! to the NI context. Findings from the current study suggest parental restrictions, being able to access and watch TV and lack of discretionary time are barriers to PA participation at home for children aged 7–9 years. Children identified access to, and high levels of, screen time negatively impacted on PA. Other important school-based barriers included a lack of space, in addition to limited or no access to equipment and facilities. The ability to have fun, play, go outside and enjoy PA were important facilitators of PA. These identified issues should be areas of focus for program developers as well as school staff to ensure that strategies are available to mitigate these factors when they serve as barriers, to support program implementation. Based on the data from the present study, many children recognized the physical health benefits of PA. However, data revealed a lack of knowledge and identified lack of PA recognition, which is comparable to previous formative research (Mackintosh et al., 2011) where some children demonstrated a limited understanding of what constitutes PA. When describing the likes and dislikes, children were more inclined to focus on their favorite activity. Participants “likes” were predominantly structured and sport-based activities were more common than play activities and feelings. Consistent with other studies (Timperio et al., 2013; Watson et al., 2015) children generally conflated PA with sport, which was confirmed within the write and draw data, with children expressing a greater recollection of structured physical activities, games (i.e., football) (refer to Figures 1A,B). In contrast, during the focus groups children ascribed PA with fun and tended to participate in PA if they perceived themselves to be physically capable; that is, they had the necessary skills and it felt it was worthwhile. Educators could teach children the meaning of PA and emphasize that it does not have to equate to sport. Providing opportunities to try different activities at school, beyond PE and organized sport, may help to redefine children's perceptions of PA to include a range of activities that they may enjoy.

Interestingly, many of the themes identified were reported to act as both facilitators and barriers across the different schools (e.g., role of parents, school environment, peer influence and interrelationships). Similar to findings from other qualitative studies, parents were perceived as both significant barriers and enablers to children's PA participation, indicating that parents have a great influence over their children's involvement in PA with the ability to both facilitate and impede participation (Brockman et al., 2009; Stenhammar et al., 2012; Noonan et al., 2016). In this study, some children specifically identified their father as a role model for PA participation (Figure 4). The findings are in line with previous research by Lijuan et al. (2017), who found that 23.3% of the variance in MVPA for boys (aged 7–11) was explained by the MVPA engagement of fathers. Sigmundová et al. (2018), found that mother's and father's levels of PA are associated with children's weekly PA. Furthermore, research suggests that active parents, who could act as role models for children, facilitate children's activity (Rodrigues et al., 2018). Consequently, targeting parents to increase their own PA levels, particularly through the participation in organized PA, may be an effective way to increase the activity levels of their children while providing health benefits for the whole family.

Within the home and family environment, our findings found that being able to go outside appeared fundamentally important to children's PA within drawings (refer to Figures 1A–D), so much so that many children reported traveling with parents to parks farther afield that are larger in size and have “better” provision. PA participation through family walks were discussed by participants alongside dog ownership. Dog ownership is associated with a number of health benefits and research indicates that children from dog-owning families accumulate more PA (Westgarth et al., 2019). Data also revealed siblings were perceived as a barrier and as a facilitator. Participants indicated that siblings may increase activity through co-participation and social support but can also persuade each other to co-participate in sedentary pursuits, including television viewing or electronic device use. Participants identified siblings' preferences for sedentary activities as a deterrent to being active, stating that the sibling was not at the same developmental level as they were in sport, in most cases mentioning that the younger sibling was not at the same level. However, participants also emphasized the positive influence siblings have on sports participation and play. Other studies looking at sibling influence on sports participation have reported similar findings (Hohepa et al., 2007; Silva et al., 2016). Kracht and Sisson (2018) reviewed siblings influence on children's objectively measured PA and found that children with siblings have a healthier PA pattern with more PA and potentially less SB. Thus, the involvement of parents, pet and/or family members appear to be fundamental to children's PA levels and it is therefore important that the key components of the intervention are structured around both parents and schools.

Whilst parents clearly have a significant role in shaping young children's health behaviors, our findings revealed that children see peer interaction as reasons to be physically active and the role of friendship groups was key in helping to promote and influence the participation of PA at home and within the school environment. Play, fun and social support were reported as key facilitators for PA participation in children aged 7–9 years. Present results are in line with previous findings that participants reported that enjoyment of PA and spending time with friends were the key influences on maintaining participation in PA (Knowles et al., 2013; Hesketh et al., 2017). Some of the enablers support previous qualitative research, including a UK qualitative study with 10–11-year-olds, which found that children engaged in PA for multiple reasons including socializing, preventing boredom and a sense of freedom (Brockman et al., 2011). Findings also support previous research which indicates that friends' social support is known to be valuable for after school activities, although parental support may be less important (Jago et al., 2009). In line with our findings, recess literature shows that playtime is a critical time to play with peers and is one of the last free-play opportunities that they have with peers (Ridgers et al., 2012; Parrish et al., 2020). Thus, providing PA opportunities that enable children to socialize with their friends was identified from focus group discussions as a key component within future intervention. Given that play (Ridgers et al., 2012), appreciation of outdoor activity (Knowles et al., 2013) and opportunities to socialize with peers (Salmon et al., 2003) are strongly associated with PA participation in children, these factors should be highlighted when planning strategies for future PA interventions. Furthermore, interventions that focus on providing social opportunities for activity may be effective activity promotion methods to control the intrinsic enjoyment of PA that was expressed by these primary-school children.

Previous systematic reviews of PA interventions in schools found favorable effects of classroom movement breaks on increasing PA (Norris et al., 2015; Martin and Murtagh, 2017; Watson et al., 2017). In this qualitative study, the majority of children found the idea of active breaks and/or incorporating movement in the classroom to be desirable. Participants perceived the additional movement as being “*very good for your body*” and suggested that the addition of activity in the classroom may have possible health benefits. However, some children noted that having to engage in active breaks or perform activities within the confined space of their classroom served as a limitation. This finding is in line with previous process evaluations exploring children's perceptions of active breaks, where some participants did not like taking part in active breaks due to lack of space (Howie et al., 2014; Watson et al., 2019). Overall, this finding suggests that researchers should consider the classroom space when adapting the *Transform-Us!* Standing workstations have the potential to be integrated in the primary-school classroom environment (Blake et al., 2012; Hinckson et al., 2013). In this study, contradictory opinions in relation to the sit-to-stand desks were apparent, with participants voicing concern over the possibility of class disruption, the practicalities and stability of the desks. Several participants mentioned potential benefits of using the sit-to-stand desks, such as improved academic performance and increased alertness. A pilot study conducted in primary schools in Australia and England, found that irrespective of implementation, integrating sit-to-stand desks into classrooms may be an effective method of reducing classroom sitting (Clemes et al., 2016). However, the acceptability and feasibility of implementing sit-to-stand desks within NI primary schools is unknown, such information is essential for determining full-scale randomized controlled trials where intervention effectiveness can be evaluated.

Data from this study reveals that several limits were placed on where and how children could play and highlights those children had little access to organized sport. In line with our results, a research study found that COVID-19 restrictions were both a barrier and a facilitator for PA in Irish adolescents (Ng et al., 2020). COVID-19 and lockdown presented opportunities with more time as a facilitator to take part in activities not done previously, like PA. Children had been working on finding new and innovative ways to remain physically active. During the period of the study, children were forced to adapt to a new “normal” due to COVID-19 restrictions, which may have caused a substantial decrease in excursions outside of the home. Recent studies highlight the decline in PA participation, with only 2.6% of children meeting the Canadian 24-Hour Movement Behaviors Guidelines, compared to rates between 12 and 17% before lockdown (Moore et al., 2020) and a three-fold reduction in PA was observed in Chinese adolescents (Xiang et al., 2020). A study conducted in Western Australia found no change in overall PA among children during the COVID-19 pandemic, and an increase in children's unstructured PA and outdoor play was observed (Nathan et al., 2021). However, as a result of the context-specific nature of the pandemic contextual factors should be taken into consideration and the imposed restrictions within and between countries, thus this finding may not be generalizable to other countries.

The weather was reported as a barrier to PA. In line with our findings, several studies have found relationships between various weather conditions and children's PA. Rainfall has been associated with decreased activity (Harrison et al., 2011; Goodman et al., 2012). Internationally, research indicates that children in Northern Europe and Melbourne, Australia were active given the weather conditions they experienced (Harrison et al., 2017). Contrary to previous research that winter weather (e.g., snow) is a barrier to outdoor playtime (Burdette et al., 2004), this research found that it was rain that hindered children. Nevertheless, it is important to note that contextual differences between North America and in the UK are apparent, and snowfall is quite infrequent and short-lived. Thus, there is a need to provide seasonal and meteorological data, when developing interventions to increase children's outdoor PA. Seasonal variation in young children's PA is apparent (Carson and Spence, 2010; McKee et al., 2012). Overcoming weather issues can be a challenge. Thus, learning to adapt to weather conditions that may provide fun opportunities for PA (i.e., wet weather) or providing parents with feasible contingency opportunities in locations where weather extremes are common, may be beneficial and may lead to consistently higher levels of PA in primary-school age children (Hesketh et al., 2017). The data also suggests that lack of time for activities is a factor that appeared to constrain family and school-based activity. Lack of time has previously been reported by youth as a significant barrier to PA (Brockman et al., 2009).

## Strengths and Limitations

This study utilized a write and draw methodology alongside focus groups, which has not been widely used in primary-school children in NI. Nevertheless, the participants in this study responded well to the combination of interactive tasks, providing important evidence for future studies to adopt a similar methodological approach. The strength of this study is the design as it includes the ability to investigate the phenomenon in natural settings and the use of a variety of research methods to obtain rich descriptions and deep insights. The drawings informed about the main environments the participants participated in PA, helped to express their opinions and experiences, and added different levels of depth to the children's responses. Triangulation of qualitative methods allowed the identification of possibly overlooked stressors, nuances in theory, and put an emphasis on the importance of context. Thus, the dual-method approach provided the participants with alternative ways to express their perceptions and experiences.

There are some limitations to this study that should be considered when interpreting the results. Due to COVID-19 protocols and curriculum restrictions, the length of the data collection was restricted, and a 45-min data collection session was stipulated by participating schools. Thus, each group completed both the write and draw task and a focus group within a 45-min timeframe, and this was standardized across all participating schools. Despite this time limitation, the results of this study offer novel findings into the experiences and understandings of PA in primary-school children, and 15–20 min write and draw activity length has been used in previous research. Furthermore, we were successful in recruiting children from several geographical locations across NI during the COVID-19 pandemic. This helps in gaining a better understanding of PA determinants in children and increases the generalizability of the study findings. It must be noted that the majority of participants were of white ethnicity and no participants had physical disabilities. Thus, future work should include a more diverse sample of participants to reflect the diversity of NI cultures. Future research should consider if another setting is more appropriate to capture children's experiences of non-school related PA, however researchers should consider the recruitment difficulties in a community setting (Brown et al., 2015).

## Conclusions

This qualitative study is the first in NI to explore the identification of barriers and facilitators of children's PA during the COVID-19 pandemic. Peers and family were main influences on children's PA and could both facilitate and impede on PA participation. Collectively these findings highlight that elements of fun, social support, time, space and are likely to be central to the adaptation and maintenance of PA among primary-school children and that activity promotion strategies that are based around co-participation with peers and family members could be an effective means of engaging in PA. Furthermore, the study findings offer suggestions to educators regarding the school and family environment. The study emphasizes social opportunities for activity may be effective activity promotion methods to provide the intrinsic enjoyment of PA that was expressed by these primary-school children.

## Data Availability Statement

The original contributions presented in the study are included in the article/Supplementary Material, further inquiries can be directed to the corresponding author/s.

## Ethics Statement

Ethical approval was granted by the Ulster University Ethics Committee (REC/20/0033). Written informed consent to participate in this study was provided by the participants' legal guardian/next of kin.

## Author Contributions

SN, AC, MM, and AG: conceptualization. SN: conducted the interviews. SN and NR: initial analysis and interpretation of the data. SN, NR, AC, MM, and AG: discussions. All authors drafted the manuscript and were part of the revision process and agreed to the final version of the paper before submission.

## Funding

SN was supported by Northern Ireland Chest, Heart and Stroke (NICHS), Belfast, UK. NR was supported by a National Heart Foundation of Australia Future Leader Fellowship (ID101895). JS was supported by a National Health and Medical Research Council Leadership Level 2 Fellowship (APP1176885). NICHS were not involved in the design of the study or collection, analysis, and interpretation of data or in writing the manuscript. No other sources of funding were used to assist in the conduct of this review or the preparation of the manuscript.

## Conflict of Interest

The authors declare that the research was conducted in the absence of any commercial or financial relationships that could be construed as a potential conflict of interest.

## Publisher's Note

All claims expressed in this article are solely those of the authors and do not necessarily represent those of their affiliated organizations, or those of the publisher, the editors and the reviewers. Any product that may be evaluated in this article, or claim that may be made by its manufacturer, is not guaranteed or endorsed by the publisher.
