# Supplementary material for: “When You Move You Have Fun”: Perceived Barriers, and Facilitators of Physical Activity From a Child's Perspective
Source: Front Sports Act Living. 2022 Mar 7;4:789259. doi: 10.3389/fspor.2022.789259 (PMC8937021; doi:10.3389/fspor.2022.789259)
Supplement: Supplementary file 1 [file Table_1.DOCX]

Supplementary Material

**Table S1.** COREQ (Consolidated criteria for Reporting Qualitative research) checklist

| **No. Item** | **Guide questions/description** | **Reported on Page #** |
| --- | --- | --- |
| **Domain 1: Research team and reﬂexivity** |  |  |
| *Personal Characteristics* |  |  |
| 1. Inter viewer/facilitator | Which author/s conducted the interview or focus group? | 5 |
| 2. Credentials | What were the researcher’s credentials? E.g. PhD, MD | 6 |
| 3. Occupation | What was their occupation at the time of the study? | 6 |
| 4. Gender | Was the researcher male or female? | 6 |
| 5. Experience and training | What experience or training did the researcher have? | 6 |
| *Relationship with participants* |  |  |
| 6. Relationship established | Was a relationship established prior to study commencement? | N/A |
| 7. Participant knowledge of the interviewer | What did the participants know about the researcher? e.g. personal goals, reasons for doing the research | 6 |
| 8. Interviewer characteristics | What characteristics were reported about the inter viewer/facilitator? e.g. Bias, assumptions, reasons and interests in the research topic | 6 |
| **Domain 2: study design** |  |  |
| *Theoretical framework* |  |  |
| 9. Methodological orientation and Theory | What methodological orientation was stated to underpin the study? e.g. grounded theory, discourse analysis, ethnography, phenomenology, content analysis | 7 |
| *Participant selection* |  |  |
| 10. Sampling | How were participants selected? e.g. purposive, convenience, consecutive, snowball | 6 |
| 11. Method of approach | How were participants approached? e.g. face-to-face, telephone, mail, email | 4/5 |
| 12. Sample size | How many participants were in the study? | 6 |
| 13. Non-participation | How many people refused to participate or dropped out? Reasons? | N/A |
| *Setting* |  |  |
| 14. Setting of data collection | Where was the data collected? e.g. home, clinic, workplace | 4/5 |
| 15. Presence of non-participants | Was anyone else present besides the participants and researchers? | 5 |
| 16. Description of sample | What are the important characteristics of the sample? e.g. demographic data, date | 4/5 |
| *Data collection* |  |  |
| 17. Interview guide | Were questions, prompts, guides provided by the authors? Was it pilot tested? | 5/6 |
| 18. Repeat interviews | Were repeat inter views carried out? If yes, how many? | N/A |
| 19. Audio/visual recording | Did the research use audio or visual recording to collect the data? | 6 |
| 20. Field notes | Were ﬁeld notes made during and/or after the interview or focus group? | N/A |
| 21. Duration | What was the duration of the inter views or focus group? | 6 |
| 22. Data saturation | Was data saturation discussed? | N/A |
| 23. Transcripts returned | Were transcripts returned to participants for comment and/or correction? | N/A |
| **Domain 3: analysis and ﬁndings** |  |  |
| *Data analysis* |  |  |
| 24. Number of data coders | How many data coders coded the data? | 7 |
| 25. Description of the coding tree | Did authors provide a description of the coding tree? | 7 |
| 26. Derivation of themes | Were themes identiﬁed in advance or derived from the data? | 7 |
| 27. Software | What software, if applicable, was used to manage the data? | 7 |
| 28. Participant checking | Did participants provide feedback on the ﬁndings? | N/A |
| *Reporting* |  |  |
| 29. Quotations presented | Were participant quotations presented to illustrate the themes/ﬁndings? Was each quotation identiﬁed? e.g. participant number | 8-11 |
| 30. Data and ﬁndings consistent | Was there consistency between the data presented and the ﬁndings? | 8-13 |
| 31. Clarity of major themes | Were major themes clearly presented in the ﬁndings? | 8-11 |
| 32. Clarity of minor themes | Is there a description of diverse cases or discussion of minor themes? | 8-11 |

**
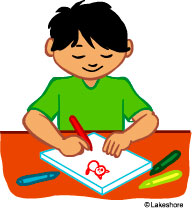
S2 Write and draw activity**

What does physical activity mean to you?

| 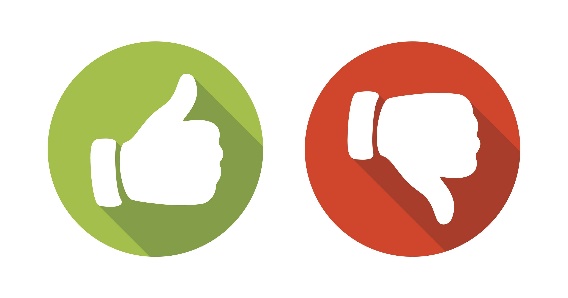What I like about physical activity | 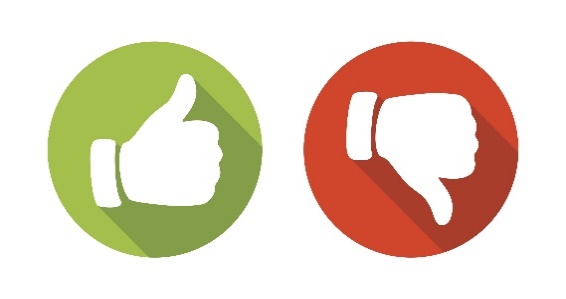What I dislike about physical activity |
| --- | --- |

If you could change your classroom, what would you change?

Where are you most likely to take part in physical activity?

Over the past week what sort of physical activities did you do outside of school?

**Table S3** Semi-structured focus group discussion guide

| **Questions** | **Focus Group Question** |
| --- | --- |
| **Introductions and icebreaker** | What is favourite physical activity and why? |
| **Photo activity** | What photo is the most active?  What photo is the least active?  What would you like to spend more time doing? |
| **Knowledge** | How much physical activity do you think you should be doing every day?  Why are some activities more active than others? |
| **School** | What is your favourite thing to play or do in your classroom?  How do you feel about using a sit-to-stand desk in your classroom? *  On school days, what do you do at breaktime?  How would you feel if your teacher sent home active homework for you to do with your parents/guardian? |
| **Family** | What stops you from doing physical activity at home?  Do you take part in any physical activity after school? |

*Including a photograph of a child working from a sit-to-stand desk.
